# Supplementary material for: Upper respiratory symptoms (URS) and salivary responses across a season in youth soccer players: A useful and non-invasive approach associated to URS susceptibility and occurrence in young athletes
Source: PLoS One. 2020 Aug 4;15(8):e0236669. doi: 10.1371/journal.pone.0236669 (PMC7402496; doi:10.1371/journal.pone.0236669)
Supplement: Attachment — Submitted filename: HZ Comments PlosOne April 2020.docx [file pone.0236669.s002.docx]

**Manuscript Number: PONE-D-20-07349**

**Title:** **Upper respiratory symptoms (URS) and salivary responses across a season in youth soccer players: a useful and non-invasive approach for predicting URS susceptibility and occurrence in young athletes**

**Reviewer' Major Comments:**

In this manuscript the authors aimed to examine the effect of a competitive season on salivary responses [cortisol (sC), testosterone (sT), Testosterone/Cortisol ratio (sT/C), Immunoglobulin A (sIgA), sIgA secretion rate (srIgA), alpha-amylase (sAA)] and upper respiratory symptoms (URS) occurrence in three teams of male soccer players (Under-15, Under-17 and Under-19 yrs.). The results show No differences were found for monthly training volume between teams. Incidence of URS was higher for the U15. Higher sT and srIgA were observed for the U19, lower sC were found for the U17 and sAA showed higher values for the U15 throughout the season. Monthly variations showed a decrease in sT in August compared to October and November for the U15. The U19 presented an increase in sC in March compared to February, sT/C were higher in March compared to February and April and sAA increased in March compared to September. Negative correlations, controlled for age group, were found between URS occurrence and srIgA (r=-0.190, p=0,001), sAA (r=-0.175, p=0.001) and sT (r=-0.115, p=0.036). The current study confirmed that youth soccer players show different age-related adaptive hormonal and immune responses to training and competition. The results show also that there is an association between training load (volume), mucosal immunity, hormone levels and URS.

The manuscript is well written and provides some new findings on this area. However, several issues must require attention.

In the **abstract** please give some significant results (e.g. values).

In the **introduction** some paragraphs must address what and why we need to know this and what the question you are addressing is and is it really that important.

In the introduction, first paragraph: which sport are the authors talking about ? soccer ?

**Methods:**

The authors have provided a detailed, yet concise, account of their study methods and experimental design. However, some precisions must be addressed:

- Please give rationality for the n values (number of participants). How did you calculate that (power analysis)?

The **discussion** reflect what the authors found, how it relates to the literature. However there is a lack of what it means physiologically and there are some speculations.

The limitations of the study may state also the cost of such follow-up and the lack of nutritional measurements.
