# Supplementary material for: Upper respiratory symptoms (URS) and salivary responses across a season in youth soccer players: A useful and non-invasive approach associated to URS susceptibility and occurrence in young athletes
Source: PLoS One. 2020 Aug 4;15(8):e0236669. doi: 10.1371/journal.pone.0236669 (PMC7402496; doi:10.1371/journal.pone.0236669)
Supplement: Attachment — Submitted filename: Response to reviewers.docx [file pone.0236669.s003.docx]

**Manuscript Number: PONE-D-20-07349**

**Title:** **Upper respiratory symptoms (URS) and salivary responses across a season in youth soccer players: a useful and non-invasive approach for predicting URS susceptibility and occurrence in young athletes**

Reviewer #1: The study presented by Lopes et al. followed a substantial number of young athletes (n=57 players aged U15 to U19) over the course of a competitive season in order to characterize changes in salivary markers of immunity and hormonal changes. They hypothesized that athletes would exhibit different humoral and immune patterns throughout the season depending on their age. Furthermore, they hypothesized that these age-specific differences would be associated with differences in incidences of Upper Respiratory Symptoms (URS). This interesting premise is based on existing literature, showing associations between salivary markers of immunity and rate of URS in young athletes (Moraes et al. 2017), adults (Fahlman et al. 2017; Guilhem et al. 2015) and elderly (Akimoto et al. 2003). The authors report differences in salivary hormonal profile, along with markers of immunity between the age-groups and throughout the season. However, the authors suggest that the measured markers of oral immunity and hormonal status could be used to predict URS in young athletes, despite finding very weak correlations between URS incidence and their outcome measures (r<0.200 for all). The authors should be careful in making bold statements that are not supported by their data. In addition, the data presented does not appear to be very novel.

Major Comments:

Associations between salivary markers of immunity and URS presented by the authors are very weak. Both appear fairly stable over time, and correlation coefficients are well below the “r=0.300” limit for “weak” associations accepted in the literature. The authors should not extrapolate their results in suggesting it could be used to predict URS. Additionally, these results should be graphically depicted to improve the overall impact of the paper.

We thank the reviewer remark and suggestion. The great novelty of this study is in the methods (the methodology). There is no other study with this kind of sampling characteristics (same local, during the same season, equal training model applied to different age groups belonging to a soccer academy). We recognize that the term predictor is not the best one, since we evaluated a possible association between variables. We altered the title, reinforcing the “association” instead of prediction. Aiming to highlight this relationship we add a graph with the relative values of URS of the three teams over the season. The figure is representative, as it gathers the markers used in the different groups, allowing to follow the trend of behaviour over time. The high interindividual variability of absolute values of this immune parameter (also founded in other studies (Walsh et al., 2011), explain the weakness of the association.

2. Many researchers have argued that decrease in sIgA and sAA concentrations may not be associated with URS, until they reach a critically low value. Per example, Neville et al. discusses the use of a 40% reduction in the athlete’s normal sIgA concentration as a threshold under which greater incidences of URS are observed. The authors describe sIgA and sAA as continuous variables, however did they also assess whether athletes reporting incidences of URS had lower than normal values of sIgA or sAA? Categorizing sAA and sIgA concentrations based on tertiles or quartiles may be strengthen their argument.

The sIgA concentration showed an elevation, of interest is that the sIgA secretion rate showed an opposite behaviour, lowering the values when more URS occurred. This is in line with Fahlman et al (2017), Moraes et al (2017) that reported a drop in the parameters of sIgA and srIga in face of cases of URS (Fahlman et al., 2017; Moraes et al., 2017). In your study, there is a drop in the first months of the season (until December – mom 5), it increases with the end of the year break in January (mom 6), and falls again until the end of the season.

3. No training intensity was available for this study, however one could hypothesize that different positions (GK vs defender vs striker etc…) may exhibit differences in the outcome measures. Did the author investigate this?

We just received the total volume of training and matches from the coaches during the study period. Unfortunately, we had no information related with the training intensity followed by the different athletes. A CV of 16% of the mean week training across a period of 10 weeks, was found in a study with a professional soccer team representing different phases of the regular competitive season (Clemente et al. 2019). In this study the seasonal variation of the volume of training and competition, is lower, showing a CV ranging from 9,7%, 10,3% and 11,3% for U15, U17 and U19 respectively. Additionally, in these soccer age groups, the weekly training schedule follows a stable predefined design, which we confirmed in an observational strategy of several microcycles of different phases of the season, which data is under the preparation of another paper. Additionally, we do not had information of the specific training loads fulfilled by players of different positions. It is a limitation of the study and we add it at the respective section in the manuscript.

4. The authors discuss the use of Testosterone/Cortisol ratio as a proxy measure of anabolic/catabolic state in the athletes. The authors collected saliva samples in the evening (18:00-19:00), where salivary cortisol was likely to be low, and thus this ratio would be more influenced by variations in sT. Furthermore sT is known to be negatively associated with overtraining (Maso et al. 2004), but it is not clear whether the authors saw a similar associations.

The circadian variation of cortisol is widely recognized, and we are aware of that. Therefore, the saliva sample was always collected at the same time, before the beginning of the first training day of the month (between 6PM and 7PM), 36 hours after the last weekend match. This time of the day is accepted in the literature as a period where cortisol shows steady values. The variation of sT showed to be trivial variation across the season, never decreasing to values, that could be associated to overtraining.

5. The result section would benefit from having more graphical depiction of the collected data. Per example, it is difficult to judge whether the changes in URS throughout the season was associated with training volume without a figure (see comment 1).

We thank the reviewer for the remark, and we add Figure 3 to highlight for each group the URS occurrence per month.

Minor Comments:

1. The authors describe that participants refrained from Caffeine and Food product; however no mention was made about alcohol consumption. Considering the differences in age amongst the participants, could this explain some of the changes seen in salivary Cortisol and Testosterone in the U19 group?

We thank the remark. Indeed, the terms of the signed commitment of all athletes of the sport academy, included under several accepted rules, the commitment to abstain from alcohol and tobacco consumption.

2. Line 308: This sentence should be reworded

We thank the remark. We rewrite the paragraph as » We observe a stable training volume in each group across de season. The CV of the total volume was 9,7%, 10,3% and 11.3% for U-15, U-17 and U-19 respectively. These CV was lower than that observed in Portuguese professional soccer players (37). Valente-dos-Santos and co-authors (38) showed that the overall training demand in soccer tends to stabilize after the player’s peak height velocity. »

3. Figure 1. Amylase is misspelled.

We apologize for the misspelled word; we made the correction.

4. Figure 1. Time effect is not represented

The effect of time is shown in Table 2 represented by the letter symbols. This was done so as not to make the graph confusing.

Reviewer #2: General:

This is an interesting paper of potential value to the field. Strengths include the long-term monitoring period (whole soccer season), and high level (national division) participants. Weaknesses include infrequent sampling time-points reducing sensitivity to observe changes across time.

The frequency of collections was monthly, always at the first week of each month, just before the first training session, assuring not less than 36 hours of recovery (from the last training session or competition. We have included this sentence in the text in the Design session.

Specific:
Abstract: recommend including some actual quantitative data/results (for main/key outcomes) in abstract results section.

We agreed with the recommendation. However, we opted for a descriptive presentation of the results in the abstract, as we think it would be the clearest way.

L78-87: Suggest change to order or mentioning these points. i.e. start with total number recruited, then number lost (dropout etc), then final number analysed.

We appreciated the suggestion and we modified in the paper.

Methods general (and subsequent results interpretation): salivary Cortisol and Testosterone are very susceptible to inaccuracy because of blood contamination in samples. It is recommended that this is screened for using biomarker assays (e.g. transferrin) because this cannot be detected visually.

Thanks for the remark. All subjects in the study were checked on a regular base for oral health by the academy medical staff. We take every care concerning to all Salimetrics indications for saliva sampling. The variation of hormonal and immune results is under expected values and in the range of the commercial kits used for analyses. When detected, some values out of the range, were excluded from analysis. Although we did not use any biomarker for blood contamination, we believe that we met all care possible

L182-187: I do not fully understand the value this metric? If you want to normalise for comparison, why not express as % of mean monthly load? e.g. October volume was 113% of the season average?

We thank the reviewer for the opportunity to clarify the presentation of the seasonal variation in the training volume observed in the 3 teams. We calculate the mean seasonal volumes (training and match) and the monthly variation around this value (Figure 1). Regarding U15 group we observed that the highest value was in October (12.3% above), whereas the U17 and U19 showed higher volumes in August, respectively 18,6% and 14,5% above de seasonal mean volume. The seasonal variation of training was low, for both teams (CV= 9,7%, 10,3% and 11,3%) for U-15, U17 and U-19, respectively.

L194: is % of total URS by age-group a fair and meaningful comparison? it would seem more appropriate to report number of episodes as % of team... or more accurately, average number of episodes per player in each age group? And also the % of players experiencing at least 1 episode and/or those experiencing no episodes during the monitoring period/each time in each age group might be useful?

We thank the remark. We now include Figure 2 with the percentage of episodes per group and in relation to the total number of episodes.

L337-338: or could it be a marker of overtraining or related to accumulated physiological stress, since OT is sometimes associated with a blunted HPA response?

We thank the remark. However, in accordance to the update concept of overtraining (OT), we are convicted that the athletes of this study should not be classified as OT (Meeusen et al., 2012), as the coincidence of psychological, immune and evident loss of performance capacity, was not detected in the teams.

L394: data not presented in results?? How does this compare to other age groups (i.e. what was the % in others) and other times of season? Any stats/sig differences?

We thank the remark. The figure 2 that we have added already allows us to verify the variation in the occurrence of URS episodes in each of the groups throughout the season and in each group in relation to the total occurrence.

L395: what about in January- in line with these points on high prevalence at this time in population?

Regarding the total number of episodes of all teams in we observed the highest values in December (n=15; 16,9%) followed by November (n=13; 14,6%). In the current season, the epidemic flu period occurred between the last week of December and the end of February, with the weekly highest incidence rate observed in the end of December. This could explain the incidence rate our study, namely regarding December. In January, 12,4% (n=11) URS episodes was also under the epidemic influence. However, for U-19 the major incidence occurred one month earlier in November (n=19%) followed by March (25%). The U-17 team showed higher incidence from September (15,2%) to December (15%) and the highest value in March (21%). The U-15 team showed a higher incidence from October to January (ranging between 18-20%). In the U17 players, 20% of the athletes showed symptoms of respiratory illness in September, despite the lower accumulation of training and the low incidence of respiratory infection symptoms in the Portuguese population (seasonal flu). We checked carefully the data and the correction was done with the added graph and in the text (see above).

L396: affection?

We apologize for the type of the word. the correct term is infection and has been changed.

L413: direct stimulating effect is too strong an assertion to make based on this design and these data... an association was observed is not the same as definite cause-effect relationship. Need to tone down language and inference here.

We thank the reviewer remark, and we followed your suggestion. We reword the paragraph. We recognize that the term predictor is not the best one, since we evaluate a possible association between variables. The term association, therefore, was added instead.

L418-419: but does need to be balanced against the desire to stimulate maximal physiological adaptation response?

Thank you for the remark. Coaches aims to stimulate under limits, the stress induction for athletes positive adaptation. However, it is necessary to keep athletes healthy to be ready to the physiological challenge of the soccer highest competitive age-group level.

L431: Then why not report the power (or Effect Size) statistics for your analyses in the results? You do mention ES in methods- this should be reported- and this will tell you if the power is OK or not.

In results section we report all effect sizes when significant differences were found. Second, in studying the impact of highly competitive youth soccer club, the limited sample size might have affected the power analysis. Removed. We calculated the a priori statistical power. For an α=0.05 and moderate effect size (*es*=0.5), the sample size of our study assures an β of 0.84 (84%). We included in the manuscript this information.

Minor typos:

L118: ...prior to saliva collection...

Thanks for the remark. We change in the text by » …before saliva collection… »

L121 3 min?

Thanks for the remark. We correct to » min »

L165: Descriptive statistics

Thanks for the remark. We fixed.

L196: "There were found a significant inverse association...." would read better as "A significant inverse association was found...."

We thank the remark and we change the text according to your suggestion.

L198: that there was a trend?

We thank the remark and we change the text. To »…showed a trend of younger players to prone to get an URS episode. »

L205: this sentence does not make sense in current form (maybe "show the pattern across time for the physiological parameters" or something similar would be better?)?

We thank the remark and the suggestion. We change the text to: » show the pattern across time for the physiological parameters »

L260: significantly?

We thank the remark and we correct the text.

L308: these data are in line with…

We thank the remark and we change the entire paragraph

[ L320: observed pattern?

L322: the same as was observed?

L326: Northern

L333: as a potential

L362: or were not selected

L379: make players more likely to experience

L389: possibly because of

L402: out of

L427: acknowledged rather than assumed? ]

We are grateful for all indications above [ … ] of spelling and typing errors by the reviewer and corrections have been made and highlighted in red in the manuscript.

Reviewer #3: Manuscript Number: PONE-D-20-07349

**Reviewer' Major Comments:**

In this manuscript the authors aimed to examine the effect of a competitive season on salivary responses [cortisol (sC), testosterone (sT), Testosterone/Cortisol ratio (sT/C), Immunoglobulin A (sIgA), sIgA secretion rate (srIgA), alpha-amylase (sAA)] and upper respiratory symptoms (URS) occurrence in three teams of male soccer players (Under-15, Under-17 and Under-19 yrs.). The results show No differences were found for monthly training volume between teams. Incidence of URS was higher for the U15. Higher sT and srIgA were observed for the U19, lower sC were found for the U17 and sAA showed higher values for the U15 throughout the season. Monthly variations showed a decrease in sT in August compared to October and November for the U15. The U19 presented an increase in sC in March compared to February, sT/C were higher in March compared to February and April and sAA increased in March compared to September. Negative correlations, controlled for age group, were found between URS occurrence and srIgA (r=-0.190, p=0,001), sAA (r=-0.175, p=0.001) and sT (r=-0.115, p=0.036). The current study confirmed that youth soccer players show different age-related adaptive hormonal and immune responses to training and competition. The results show also that there is an association between training load (volume), mucosal immunity, hormone levels and URS.

The manuscript is well written and provides some new findings on this area. However, several issues must require attention.

In the **abstract** please give some significant results (e.g. values).

We agreed with the recommendation. However, we opted for a descriptive presentation of the results in the abstract, as we think it would be the clearest way.

In the **introduction** some paragraphs must address what and why we need to know this and what the question you are addressing is and is it really that important.

This study applies for the use of a scientific approach to the training organization of youth soccer. Moreover, will permit a better understanding of the load dynamics in the last three age-groups of the youth department and, as well, the age-groups where the game has is formal format (eleven vs. eleven). With this in mind it’s possible to the coaches and researchers to highlight the mechanisms that are underpinning the training sessions and, by inference, the competition, putting all in line with the progressive demands of soccer talent development programs. We have already included this paragraph in the introduction.

In the introduction, first paragraph: which sport are the authors talking about ? soccer ?

We included the term soccer in introduction.

**Methods:**

The authors have provided a detailed, yet concise, account of their study methods and experimental design. However, some precisions must be addressed:

- Please give rationality for the n values (number of participants). How did you calculate that (power analysis)?

The participants were players of the youth department of a professional club and the sample is composed by the total number of the players of the mentioned age-groups (Under-15, Under-17 and Under-19). We calculated the a priori statistical power. For an α=0.05 and moderate effect size (*es*=0.5), the sample size of our study assures an β of 0.84 (84%).

The **discussion** reflect what the authors found, how it relates to the literature. However there is a lack of what it means physiologically and there are some speculations.

With our results we were able to show that the groups have a different immune and hormonal response.The monitoring of sIgA, testosterone and α-amylase levels could provide a useful and non-invasive approach associated to URS susceptibility and occurrence in young athletes during an entire soccer season, helping detect those more prone to illness. With this knowledge, coaches can potentially manipulate daily training loads to attenuate the physical stressors imposed upon the athletes and thereby decrease the likelihood of URS occurrence, especially at more demanding and stressful periods. In considering that in youth soccer players the prevalence of URS is higher with respect to the seasonal influence of severe weather conditions, coaches are urged to consider the effect of accumulation of training load and matches on the athletes’ immune response.

The limitations of the study may state also the cost of such follow-up and the lack of nutritional measurements.

**References**

Fahlman, M., Engels, H., Hall, H., 2017. SIgA and Upper Respiratory Syndrome During a College Cross Country Season. Sport. Med. Int. Open. https://doi.org/10.1055/s-0043-119090

Moraes, H., Aoki, M.S., Freitas, C.G., Arruda, A.F.S., Drago, G., Moreira, A., 2017. SIgA response and incidence of upper respiratory tract infections during intensified training in youth basketball players. Biol. Sport. https://doi.org/10.5114/biolsport.2017.63733

Walsh, N., Gleeson, M.M., Shephard, R., 2011. Position statement part one: immune function and exercise. Exerc. Immunol. Rev. 17, 6–63.
